# Supplementary figures and images for: CD28 shapes T cell receptor signaling by regulating Lck dynamics and ZAP70 activation
Source: Front Immunol. 2024 Dec 24;15:1503018. doi: 10.3389/fimmu.2024.1503018 (PMC11703918; doi:10.3389/fimmu.2024.1503018)

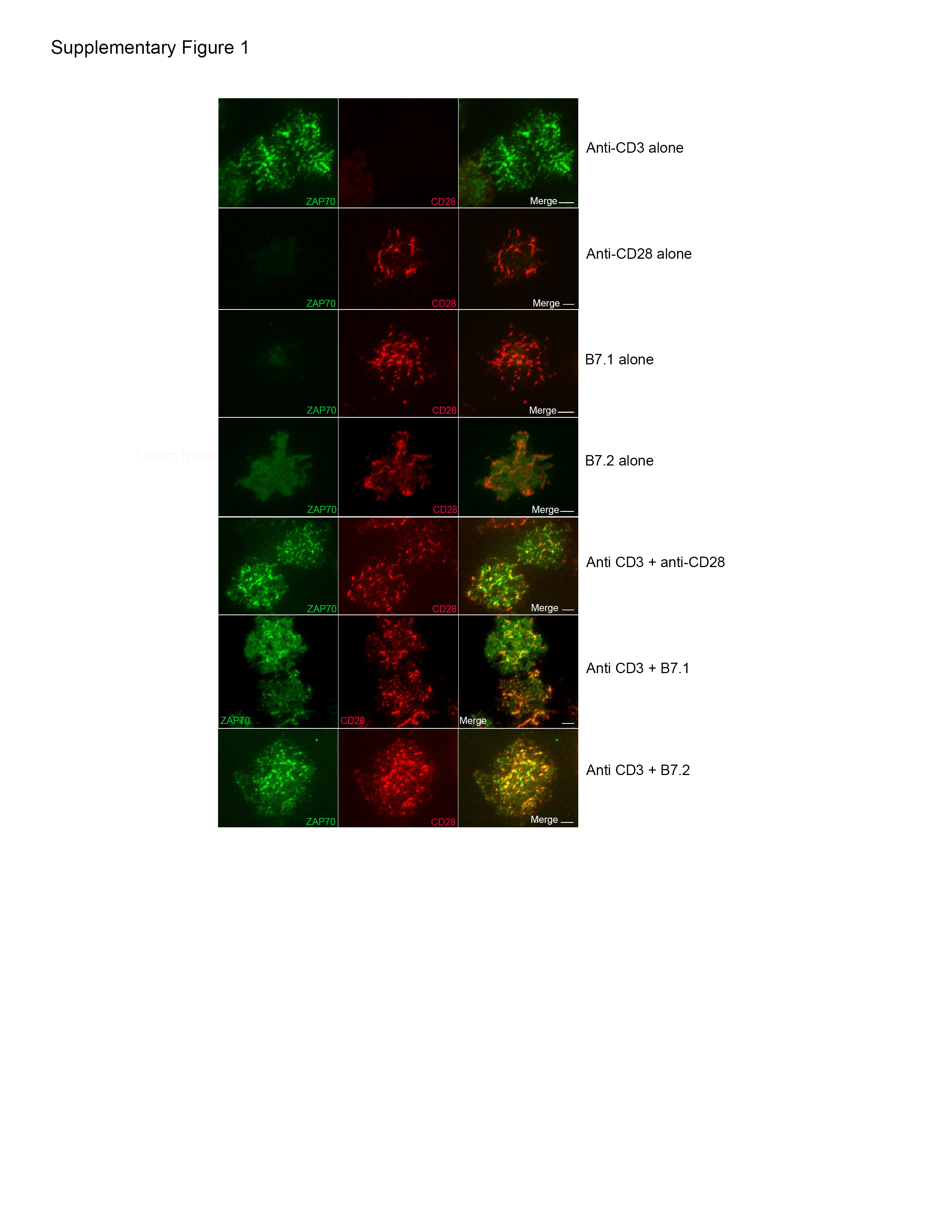

Supplement: Supplementary Figure 1 — Anti-CD28 antibody and natural ligands for CD28 (B7.1 and B7.2) specifically form CD28 microclusters which colocalize with ZAP70. Jurkat T cells were transfected to express ZAP70-Emerald (green) and CD28-Apple (red). Cells were activated on coverslips coated with indicated antibodies, natural ligands, or combinations. Images were acquired at 21°C using TIRF microscope. Scale bar: 5µm. [file Image1.jpeg]

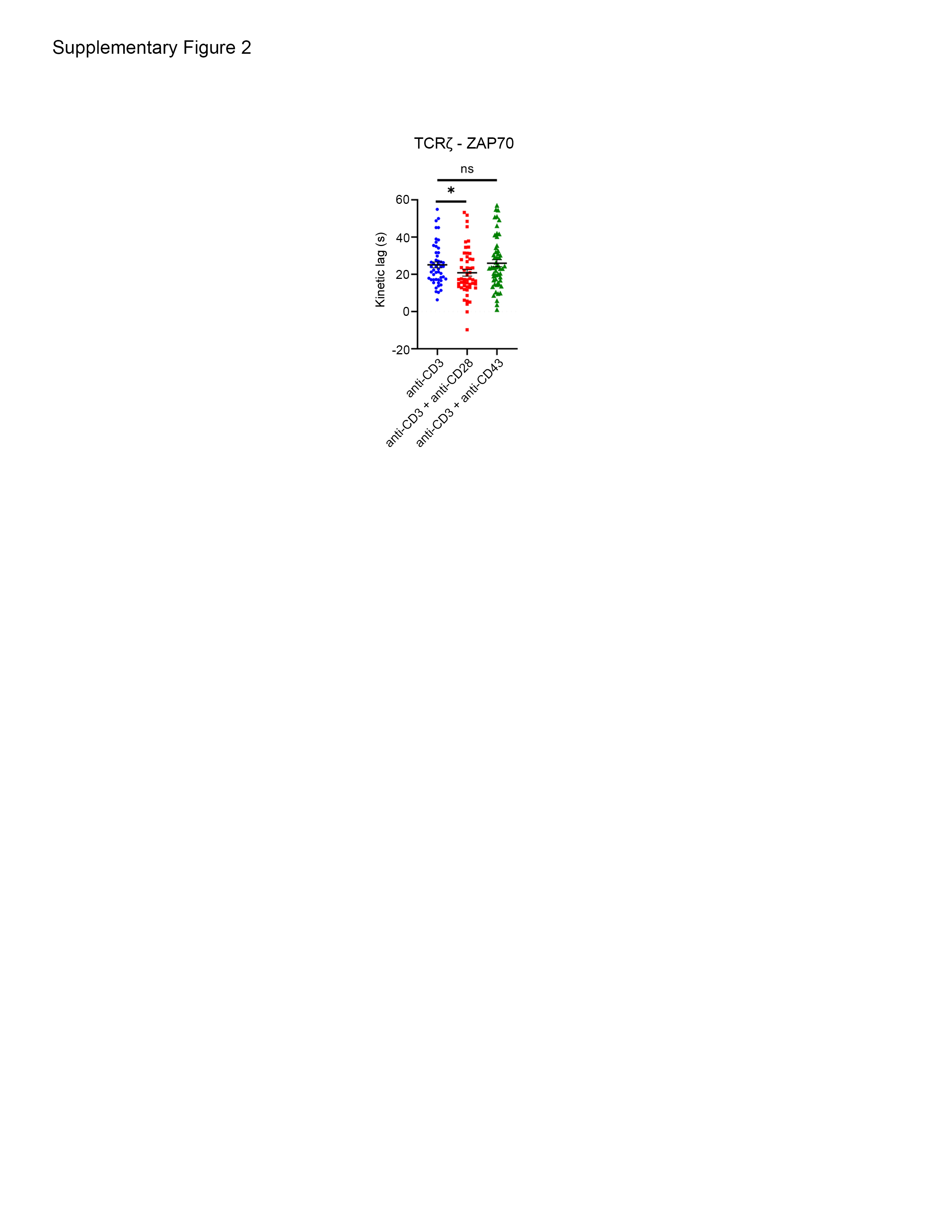

Supplement: Supplementary Figure 2 — Accelerated recruitment of ZAP70 to TCRζ in the TCR microclusters is specifically driven by anti-CD28 antibody. Jurkat T cells were transfected to express TCRζ-Emerald (green) and ZAP70-Apple (red) and were activated on coverslips coated with either anti-CD3 alone (n = 50 MC, >7 cells) or with anti-CD3 + anti-CD28 (n = 52 MC, >7 cells) or with anti-CD3 + anti-CD43 (n = 55 MC, >7 cells). 120 time-lapse images were acquired every 3s at 21°C using TIRF microscope. Kinetic lags measured between TCRζ and ZAP70 with indicated stimulatory antibodies. Data presented as mean ± SEM. Populations were analyzed using Mann-Whitney tests, *p < 0.05, **p < 0.01. [file Image2.jpeg]

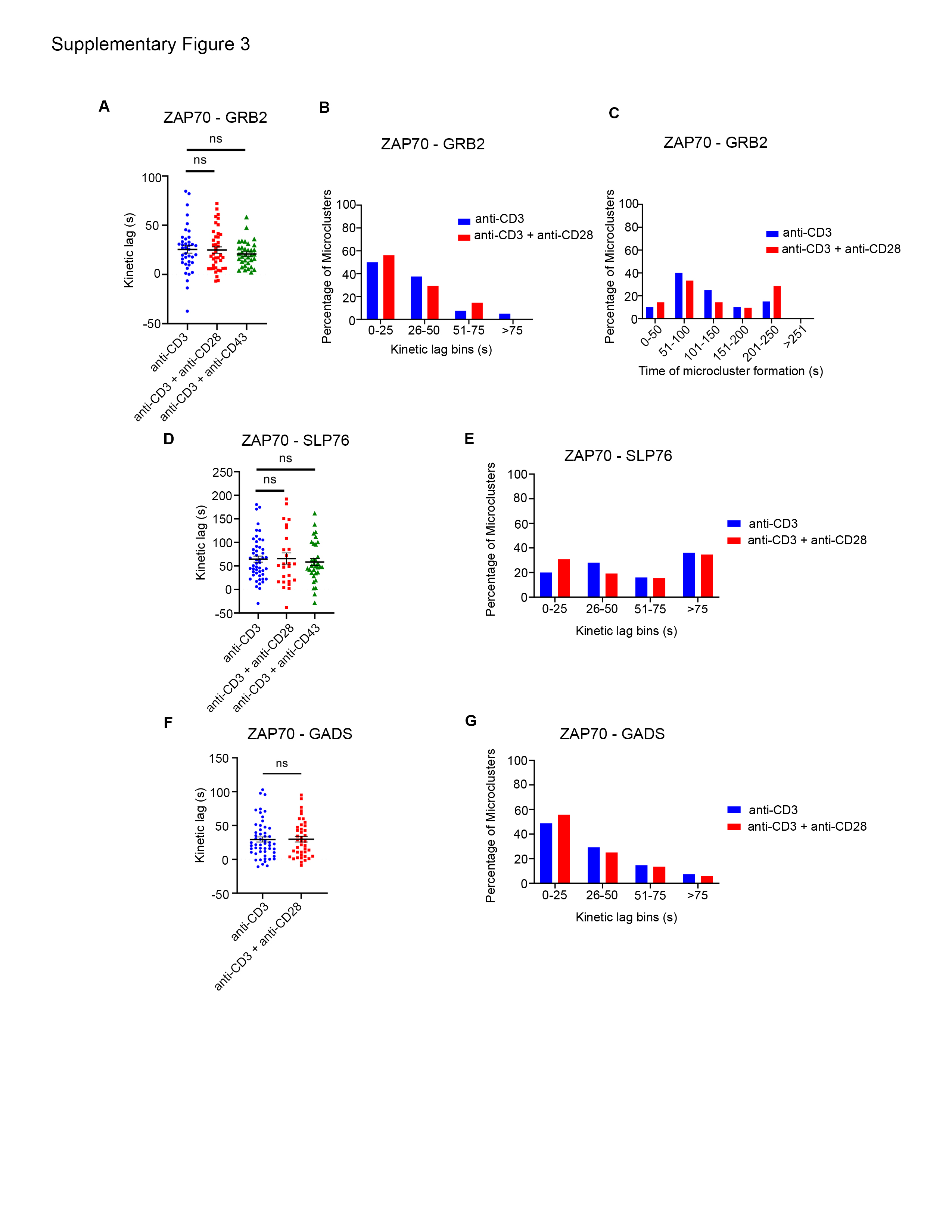

Supplement: Supplementary Figure 3 — Effect of CD28 co-stimulation is less pronounced in regulating kinetic lag between ZAP70 and signaling domain proteins than kinetic lag between TCRζ and ZAP70. Jurkat T cells were transfected to express ZAP70-Apple (red) and GRB2-Emerald (green) (A–C) or ZAP70-Apple (red) and SLP76-Emerald (green) (D, E), or ZAP70-Apple (red) and GADS-Emerald (green) (F, G) and were activated on coverslips coated with either anti-CD3 alone (ZAP70-GRB2 lag: n = 40 MC, >8 cells; ZAP70-SLP76 lag: n = 50 MC, >9 cells; ZAP70-GADS lag: n = 53 MC, >11 cells), or with anti-CD3 + anti-CD28 (ZAP70-GRB2 lag: n = 41 MC, >11 cells; ZAP70-SLP76 lag: n = 26 MC, >6 cells; ZAP70-GADS lag: n = 41 MC, >9 cells), or with anti-CD3 + anti-CD43 (ZAP70-GRB2 lag: n = 35 MC, >5 cells; ZAP70-SLP76 lag: n = 33 MC, >5 cells). 120 time-lapse images were acquired every 3s at 21°C using TIRF microscope. (A, D, F) Kinetic lags measured between ZAP70-GRB2, ZAP70-SLP76, and ZAP70-GADS with indicated stimulatory antibodies. (B, E, G) Distribution of average kinetic lags across specified time bins for indicated proteins. (C) Distribution of percentage of microclusters formed at specified time intervals of the first kinetic lag bin (0-25s) in (B). Data presented as mean ± SEM. Populations were analyzed using Welch’s t-tests (ZAP70-GRB2 lag) and Student’s t-tests (ZAP70-SLP76 lag), Mann-Whitney test (ZAP70-GADS lag), *p < 0.05, **p < 0.01. [file Image3.jpeg]

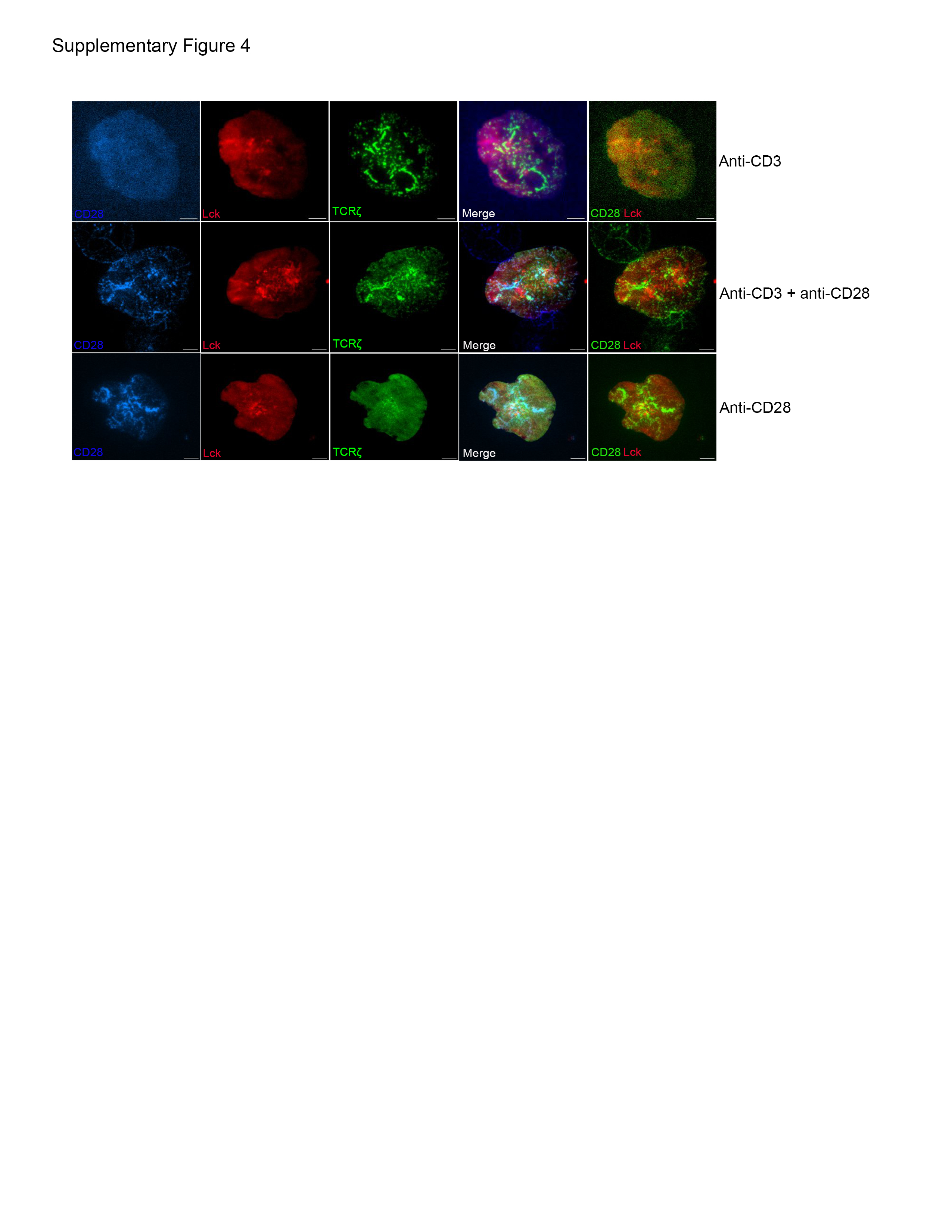

Supplement: Supplementary Figure 4 — Recruitment of CD28 alone can lead to formation of Lck clusters that colocalize with TCRζ. Jurkat T cells were transfected to express CD28-Turquoise (blue), Lck-Apple (red) and TCRζ-YFP (green) and were activated on coverslips coated with either anti-CD3 alone, or with anti-CD3 + anti-CD28, or with anti-CD28 alone. 120 time-lapse images were acquired every 3s at 21°C using TIRF microscopy. Scale bar: 5µm. [file Image4.jpeg]

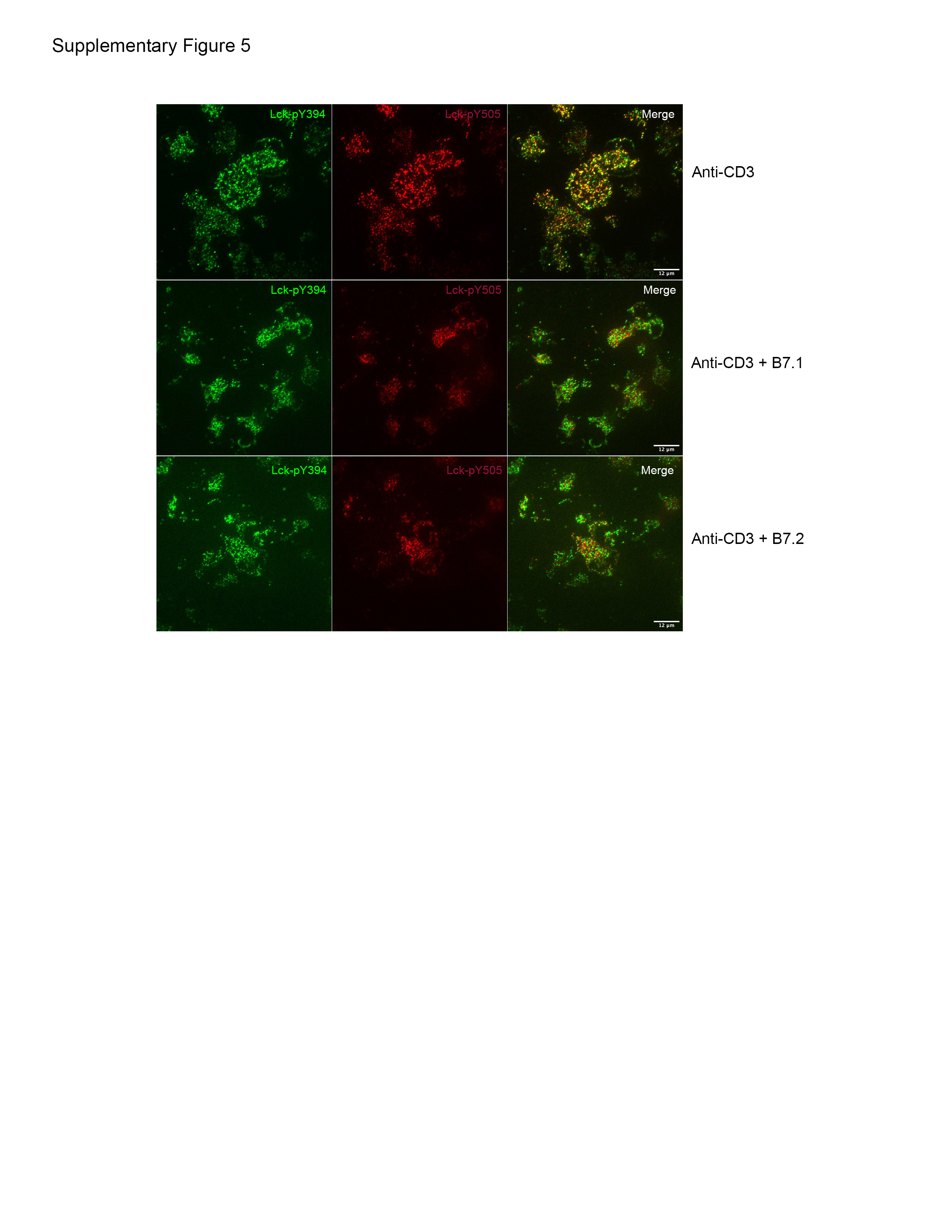

Supplement: Supplementary Figure 5 — CD28 co-stimulation leads to greater separation between Lck-pY395 and Lck-pY505. Jurkat T cells were stimulated with anti-CD3 alone, anti-CD3 + B7.1 and anti-CD3 + B7.2 coated coverslips for 5 minutes and then stained with Lck-pY395 and Lck-pY505. Images were acquired using TIRF microscopy. Scale bar is 12 µm. [file Image5.jpeg]
